# Two-Dimensional Convolutional Neural Network for Depression Episodes Detection in Real Time Using Motor Activity Time Series of Depresjon Dataset

**DOI:** 10.3390/bioengineering9090458

**Published:** 2022-09-09

**Authors:** Carlos H. Espino-Salinas, Carlos E. Galván-Tejada, Huizilopoztli Luna-García, Hamurabi Gamboa-Rosales, José M. Celaya-Padilla, Laura A. Zanella-Calzada, Jorge I. Galván Tejada

**Affiliations:** 1 Unidad Académica de Ingeniería Eléctrica, Universidad Autónoma de Zacatecas, Jardín Juarez 147, Centro, Zacatecas 98000, Mexico; 2 Consejo Nacional de Ciencia y Tecnología, Universidad Autónoma de Zacatecas, Jardín Juarez 147, Centro, Zacatecas 98000, Mexico; 3 LORIA (INRIA, CNRS), Université de Lorraine, Campus Scientifique BP 239, 54506 Nancy, France

**Keywords:** depression, motor activity, convolutional neural network, depressive episodes, artificial intelligence

## Abstract

Depression is a common illness worldwide, affecting an estimated 3.8% of the population, including 5% of all adults, in particular, 5.7% of adults over 60 years of age. Unfortunately, at present, the ways to evaluate different mental disorders, like the Montgomery–Åsberg depression rating scale (MADRS) and observations, need a great effort, on part of specialists due to the lack of availability of patients to obtain the necessary information to know their conditions and to detect illness such as depression in an objective way. Based on data analysis and artificial intelligence techniques, like Convolutional Neural Network (CNN), it is possible to classify a person, from the mental status examination, into two classes. Moreover, it is beneficial to observe how the data of these two classes are similar in different time intervals. In this study, a motor activity database was used, from which the readings of 55 subjects of study (32 healthy and 23 with some degree of depression) were recorded with a small wrist-worn accelerometer to detect the peak amplitude of movement acceleration and generate a transient voltage signal proportional to the rate of acceleration. Motor activity data were selected per patient in time-lapses of one day for seven days (one week) in one-minute intervals. The data were pre-processed to be given to a two-dimensional convolutional network (2D-CNN), where each record of motor activity per minute was represented as a pixel of an image. The proposed model is capable of detecting depression in real-time (if this is implemented in a mobile device such as a smartwatch) with low computational cost and accuracy of 76.72% In summary, the model shows promising abilities to detect possible cases of depression, providing a helpful resource to identify the condition and be able to take the appropriate follow-up for the patient.

## 1. Introduction

Information from World Health Organization (WHO) states that depression is a common disease worldwide, affecting approximately 280 million people, and is characterized by abrupt mood changes and emotional responses to problems in daily life. When it is recurrent and with moderate or severe intensity, depression may become a serious health condition. The appearance of depressive symptoms can cause great suffering to the affected person and disrupt work, school, and family activities. In the worst cases, it can lead to suicide. More than 700,000 people commit suicide every year. Suicide is the fourth leading cause of death in the 15–29 age group [1]. The lifetime prevalence of depression is about 15% [2], but the incidence of episodes with a severity level not qualifying for a depressive diagnosis is far more prevalent [3,4]. According to Berenzon et al. [5] it is a recurrent mental disorder that usually begins at an early age and causes a gradual reduction in functioning, resulting in large economic and social costs, which is why depression is at the top of the list of disabling conditions. Depression, in the worst cases, can lead to suicide in people who suffer from it. It is even the second leading cause of death, as according to WHO data about 800,000 people commit suicide each year [1].

In the last two years, depression prevalence was susceptible to change as the COVID-19 pandemic caused in many ways overwhelming situations for the whole world. Some of these situations were very obvious, such as COVID-19-derived illnesses, financial stability, loss of work; death of family, friends, partners; and quarantine, especially for those living alone. Combating depression problems in situations like these where isolation is mandatory is a much more difficult task to deal with than face to face with qualified mental health professionals for those who need it [6].

The problems related to the situation of not being able to attend to patients with a certain degree of depression in person are also added. Besides, ways to evaluate different mental disorders need a great effort on part of specialists due to the lack of availability of patients for obtaining the necessary information. This means that it largely depends on the patient’s efforts to cooperate in communicating their symptoms and problems [7].

The main alternative to knowing the level of depression of patients is known as the Montgomery–Asberg Rating Scale (MADRS), which was developed in the late 1970s [8]. Specialists rate 10 depression-relevant items based on observations and a discussion with the patients, the sum score depends on the range in which they are found, and the severity of the case will be determined. If the score is less than 10 it indicates that there are no symptoms of depression [9], and a score greater than 30 indicates a certain degree of depression [10]. However, since this score remains subjective to the discussion with the patient, a system focused on the biological signals that the body produces is necessary to have a more objective assisted diagnosis that serves as support to the clinicians in charge of treating conditions such as depression.

To address this type of problem, some research has focused its efforts on the study of biological signals and deep learning algorithms to detect early and objectively symptoms of depression. In the case of Sandheep et al. [11], a deep learning method is used, where a convolutional neural network (CNN) was trained using electroencephalogram (EEG) signals from 60 persons (30 normal and 30 depressed). The network attained the highest accuracy of 99.31% in classifying depression. Other works have had interesting findings using electrical activity produced by the brain, such as Mumtaz et al. [12], who proposed an EEG-based deep learning framework that automatically classifies depressed and healthy subjects. The classification results involving the CNN model had an accuracy of 98.32%, concluding that deep learning could be used for depression detection. Besides, Li et al. [13] used a CNN to classify mental load, and they merged the functional connectivity matrices from three EEG bands that performed the best into a three-channel image to classify mild depression-related and normal EEG signals using the CNN. The results for the classification model obtained an accuracy of 80.74%, and the method showed that coherence, correlation, and the phase-locking value can effectively recognize mild depression using CNN. Finally, one of the most recent studies where the main data is generated by EEG signals is that of Sarkar et al. [14], who sought to design and develop an expert system by exploiting the hidden power of various deep learning (DL) and machine learning (ML) techniques to track mental depression from EEG data. Among neural network-based deep learning techniques, the recurrent neural network model has achieved the highest accuracy with 97.50% in the training set and 96.50% in the testing set.

While each of the aforementioned studies present relevant results and a contribution to the field of mental health, the nature of these studies implies being—to some extent—invasive to a patient, which may cause a significant bias in the data extraction—not to mention that their implementation in different real environments would prove to be a problem, since at the moment it is not possible to measure these signals without special equipment.

Although some research investigations on the identification of depression are based on audiovisual cues [15]. Others implement more discreet methods, such as Amanant et al. [16], who proposed to implement a long-short term memory (LSTM) model to predict depression from text, semantics, and written content. The proposed framework achieves 99.0% accuracy. While, Kour [17] proposed a hybrid model for depression prediction using two deep learning architectures, CNN and bi-directional long short-term memory (biLSTM), which obtained after optimization an accuracy of 99.28% on benchmark depression-containing tweets. The results showed a profound difference between the linguistic representation of depressive and non-depressive content.

Nowadays, portable devices are part of the daily life of almost any person. These devices have elements that make physical and mental monitoring possible, which also makes it possible to collect a large amount of data every day to monitor their physical condition and even measure the quality of life. This means that even the data extracted by these devices can be used to detect various mental health problems such as depression [18]. One thing that characterizes human beings is indisputably the physical movements we make during the day. This can be described as motor activity, which is nothing more than repeated social rhythms in interaction with biological rhythms, driven by the 24-h circadian clock intertwined with numerous 2 to 6-h ultradian cycles [19]. If these biological rhythmic patterns are out of sync, there may be significant symptoms of mood disturbance [20]. Actigraphs are non-invasive devices capable of monitoring rest and human activity cycles that typically record gravitational acceleration units through a wristband [21].

One of the devices that have been used for the collection of motor activity data is the actigraph. Several research works have used these data to generate models for the classification, detection, and supervision of this illness using artificial intelligence techniques. One recent study proposal by García-Ceja et al. [22] presented the analysis of a single dataset containing actigraphies of patients suffering from depression, where they applied machine learning to classify patients into depressed and non-depressed with an F1-Score of 0.73, and the findings showed the data contains information that can be used to determine a person’s state of depression. On the other hand, Zanella-Calzada et al. [23] proposed an analysis of motor activity to find a relationship between a series of statistical features, based on continuous values acquired at a specific time and possible depression. Although the results obtained were favorable with an accuracy of 89% using random forest, the main limitation of this study is the large volume of data on the motor activity required by each patient. Galván-Tejada et al. [24] studied the motor signals generated through smart bands, obtained from the “Depresjon” database. They carried out a statistical feature extraction approach, in time and spectral evolution of the signal, to later implement an intelligent selection of features using genetic algorithms to reduce the amount of information required to provide a rapid non-invasive diagnosis with an area under the curve of 0.73, thus concluding that it is possible to distinguish between depressive states almost in real-time. However, the number of processes to be carried out to obtain the necessary information for its analysis ends up being one of its possible limitations.

Additionally, the use of motor activity in time series has been used to acquire information that helps identify possible cases of depression. Frogner et al. [25] developed a one-dimensional convolutional neural network (1D-CNN) to detect depression through measurements of motor activity, where three models with different time segments and different functions were trained. The first model classifies the participants into a condition group and a control group. In the following experiment, another model was trained to classify the level of depression of the participants, and, finally, a third model was trained to predict the MADRS scores, obtaining an average F1-Score of 0.7, as a result, to detect the control and condition groups and 0.30 to detect levels of depression. The prediction of the MADRS scale gave the result a root mean square error of 4.0. Rodríguez-Ruiz et al. [26] developed classification models with three-time series or three different subsets (day, night and full day) to detect models of depression based on motor activity and in the extraction of 24 characteristics in the time and frequency domain to select the best model to be used in their classification of depression episodes. Their results showed that the best subset of data for the detection of depressive states is the signals of nocturnal motor activity with an accuracy of 99.7%. Therefore, they were able to conclude that it is possible to identify subjects with the presence of depression from its developed model. In the work of Jakobsen et al. [27], their objective was to investigate whether objective signals of motor activity can help current diagnosis, through the application of machine learning techniques and thus by analyzing patterns of activity in depressed patients and healthy controls. They used different algorithms such as Random Forest, Deep Neural Network, and Convolutional Neural Network to analyze 14 days of motor activity recorded with an active watch. The statistical characteristics analyzed were the mean activity, the standard deviation of the mean activity, and the proportion of zero activity where they obtained an accuracy of 84%, applying class balancing and Deep Neural Networks.

Since the behavior of each person is non-stationary, it is important to use adaptive artificial intelligence models for depression recognition. The aim of this work is to present a method capable of objectively detecting episodes of depression through a two-dimensional convolutional neural network (2D-CNN), using a small amount of motor activity data to classify different types of patients. In different environments it can be implemented in small portable devices with no high level of processing.

The paper is organized as follows: Section 2 details the materials and methods used and the methodology proposed. In Section 3, the results obtained through the 2D-CNN model and its validation are presented Section 4 discusses these results and compares them to those presented in the state of the art. In Section 5 we provide the conclusions.

## 2. Materials and Methods

**Figure 1:**
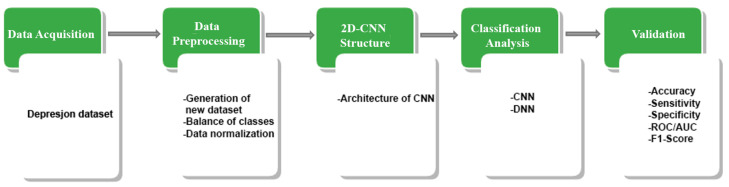
Flowchart of the methodology proposed. The green squares refer to data processing methodology while the white squares detail the task involved in each step.

The implemented methodology in this investigation is summarized in five stages, each of which is presented in Figure 1. The first step indicates the source from which the motor activity record of participants or patients is obtained. The second stage, which is data pre-processing, explains in detail how a new setup of a dataset is generated from the original data. This stage also includes the use of a technique to solve the class imbalance present in the data, to subsequently apply a normalization. The third stage consists of modeling a 2D-CNN architecture, where the number of convolutional layers, filter size (used for feature extraction), pooling, number of epochs, batch size, and activation function used are explained. Then, the analysis classification is performed through the proposed convolutional neural network and deep neural network with different depth levels to explore the difference between one network and the other. To finish in the validation stage, the different classification models are evaluated using metrics such as receiver operating characteristic (ROC) curve, area under the curve (AUC), accuracy (ACC), and F1-Score.

### 2.1. Data Description

The dataset used for the development of this research is the Depresjon dataset [4], a publicly available dataset that collects motor activity with a wrist worn accelerometer device. This device constitutes of a rectangular piezoelectric bimorph plate and seismic mass. It is omnidirectional, but is most sensitive in the vertical axis. This active watch technology detects the peak amplitude of movement acceleration and generates a transient voltage signal proportional to the rate of acceleration. The device has a sampling frequency of 32 Hz and collects motion in the frequency range of 0.5–7.0 Hz [21].

The dataset consists of actigraph data collected from 23 unipolar and bipolar depressed patients (condition group) at the Haukeland University Hospital. Five subjects were hospitalized during their data collection period and 18 were outpatients. The severity level of the ongoing depression was rated by a clinician on the MADRS [8]. The dataset also contains the actigraphy data from 32 healthy control persons, consisting of 23 hospital employees, 5 nursing students, and 4 healthy persons recruited from a general practitioner. None had a history of either psychotic or effective disorders.

For this work, only the data collected over time is used, including the features “timestamp” (one-minute intervals), “date” (date of measurement), and “activity” (activity measurement from the actigraph watch).

### 2.2. Data Pre-Processing

The data pre-processing consists of three main steps, the generation of the new dataset, the balance of classes, and data normalization.

First, a period of a week (in intervals of one minute) is taken from the original data to generate a new dataset containing enough observations to be processed through a convolutional neural network. This is to determine that in this period it is enough to create a classifier that can identify cases of depression, keeping the network simple without much depth.

The motor activity data (MAD) of each study subject are stored in columns $C_{ij}$, containing the MAD value obtained per minute during a time range from 00:00 to 23:59 (one day). These columns are transposed to become a vector $V^{T}$. For each subject, both depressed and not depressed, their vector is extracted per day until completing 7 days (one week), which is added to matrix *A*. To better understand the columns composition, given the MAD values of participant 1 on days 1, 2, 3, 4, 5, 6, and 7, the columns obtained correspond to $C_{1,1},C_{1,2},C_{1,3},C_{1,4},C_{1,5},C_{1,6},C_{1,7}$, each containing 1440 arrows (60 min multiplied by 24 h). This is represented in Equation (1).

(1) $$\begin{array}{c} C_{11}=\begin{array}{c} \mathrm{Min}.\\ 00:00\\ 00:01\\ 00:02\\ 00:03\\ 00:04\\ \ldots \\ 23:59 \end{array}\begin{array}{c} \mathrm{MAD}\\ \left[\begin{array}{c} 168\\ 107\\ 550\\ 157\\ 0\\ \ldots \\ 208 \end{array}\right] \end{array}C_{12}=\begin{array}{c} 00:00\\ 00:01\\ 00:02\\ 00:03\\ 00:04\\ \ldots \\ 23:59 \end{array}\begin{array}{c} \left[\begin{array}{c} 168\\ 17\\ 560\\ 167\\ 23\\ \ldots \\ 280 \end{array}\right] \end{array}C_{13}=\begin{array}{c} 00:00\\ 00:01\\ 00:02\\ 00:03\\ 00:04\\ \ldots \\ 23:59 \end{array}\begin{array}{c} \left[\begin{array}{c} 118\\ 157\\ 450\\ 157\\ 0\\ \ldots \\ 305 \end{array}\right] \end{array}C_{14}=\begin{array}{c} 00:00\\ 00:01\\ 00:02\\ 00:03\\ 00:04\\ \ldots \\ 23:59 \end{array}\begin{array}{c} \left[\begin{array}{c} 148\\ 157\\ 120\\ 457\\ 121\\ \ldots \\ 892 \end{array}\right] \end{array}\\ C_{15}=\begin{array}{c} 00:00\\ 00:01\\ 00:02\\ 00:03\\ 00:04\\ \ldots \\ 23:59 \end{array}\begin{array}{c} \left[\begin{array}{c} 168\\ 107\\ 550\\ 157\\ 0\\ \ldots \\ 208 \end{array}\right] \end{array}C_{16}=\begin{array}{c} 00:00\\ 00:01\\ 00:02\\ 00:03\\ 00:04\\ \ldots \\ 23:59 \end{array}\begin{array}{c} \left[\begin{array}{c} 168\\ 17\\ 560\\ 167\\ 23\\ \ldots \\ 280 \end{array}\right] \end{array}C_{17}=\begin{array}{c} 00:00\\ 00:01\\ 00:02\\ 00:03\\ 00:04\\ \ldots \\ 23:59 \end{array}\begin{array}{c} \left[\begin{array}{c} 118\\ 157\\ 450\\ 157\\ 0\\ \ldots \\ 305 \end{array}\right] \end{array} \end{array}$$

At the same time, its output value is assigned (0 = Not depressed, 1 = Depressed) and added to matrix *A*. It continues with the next subject until completing the 55 participants, as shown in Equation (2).

(2) $$A=\begin{array}{cccccccccc} \; & 00:00 & 00:01 & 00:02 & 00:03 & 00:04 & \; & 23:59 & Output\\ V_{1,1}^{T}= & [168 & 107 & 550 & 157 & 0 & \ldots & 208] & 0\\ V_{1,2}^{T}= & [168 & 17 & 560 & 167 & 23 & \ldots & 280] & 0\\ V_{1,3}^{T}= & [118 & 157 & 450 & 157 & 0 & \ldots & 305] & 1\\ V_{1,4}^{T}= & [148 & 157 & 120 & 457 & 121 & \ldots & 892] & 1\\ \; & \; & & \; & \ldots & \; & & \; & & \\ V_{55,6}^{T}= & [15 & 566 & 486 & 102 & 452 & \ldots & 302] & 0\\ V_{55,7}^{T}= & [563 & 124 & 456 & 321 & 265 & \ldots & 201] & 1 \end{array}$$

Since the number of not depressed instances is greater than the number of depressed instances, a process of class balancing is applied using the Adaptative Synthetic (ADASYN) sampling technique. This technique consists of using a weighted distribution for different minority class examples, according to their level of difficulty in learning, where more synthetic data is generated for minority class examples that are easier to learn [28].

The calculation of the degree of class imbalance can be done with Equation (3), where $m_{s}$ and $m_{l}$ are the number of minority class examples and the number of majority class examples, respectively, and *d* ϵ (0,1].

(3) $$d=m_{s}/m_{l}$$

If $d<d_{th}$ ($d_{th}$ is a preset threshold for the maximum tolerated degree of class imbalanced ratio), the number of synthetic data examples that need to be generated for the minority class can be calculated with Equation (4).

(4) $$G=(m_{s}\;-m_{l})\;\times \;\beta$$

where β ϵ [0,1] is a parameter used to specify the desired balance level after the generation of the synthetic data. β = 1 refers to a fully balanced dataset.

For each example $x_{i}$ ϵ [minority class], find *K* nearest neighbors based on the Euclidean distance in *n* dimensional space and calculate the ratio $r_{i}$ defined in Equation (5).

(5) $$r_{i}=\Delta /K,i=1,\ldots ,m_{s}$$

where $\Delta_{i}$ is the number of examples in the *K* nearest neighbors of $x_{i}$ that belong to the majority class. Therefore, $r_{i}$ ϵ [0,1].

For the next step, a normalization $r_{i}$ is applied, using Equation (6).

(6) $${\hat{r}}_{i}=r_{i}/\sum_{i=1}^{m_{s}}r_{i}$$

where ${\hat{r}}_{i}$ is a density distribution, shown in Equation (7).

(7) $$\left(\sum_{i}^{}{\hat{r}}_{i}=1\right)$$

Then, the calculation of the number of synthetic data examples that need to be generated for each minority example $x_{i}$ is calculated using Equation (8).

(8) $$g_{i}={\hat{r}}_{i}\times G$$

where *G* is the total number of synthetic data examples that need to be generated for the minority class, as defined in Equation (9). For each minority class data example $x_{i}$, $g_{i}$ synthetic data examples are generated according to the next steps.

First, we do the loop from 1 to $g_{i}$. Then, we randomly choose one minority data example, $x_{zi}$, from the *K* nearest neighbors for $x_{i}$. Finally, we generate the synthetic data example according to Equation (9).

(9) $$s_{i}=x_{i}+\left(x_{zi}-x_{i}\right)\times \lambda$$

where $(x_{zi}-x_{i}$) is the difference vector in *n* dimensional spaces, and λ ϵ [0,1] is a random number.

A final step of normalization is applied using the Equation (10), where $z_{i}$ represents the current value normalized, $x_{i}$ represents the original value, μ represents the mean of the column where the value is located and σ represents the standard deviation. This step is performed to avoid overfitting problems.

(10) $$z_{i}=\frac{x_{i}-\bar{x}}{\sigma }$$

### 2.3. 2D-CNN Structure

Table 1 shows the 2D-CNN structure adopted by this research. Input data images were resized to 30 × 48 to be consistent with the number of data for each study subject. The height and width were calculated based on the 1440 data points representing the motor activity. The CNN model consists of three convolutional layers, two pooling layers, and three fully connected layers (FLC).

The convolutional layer generates new data called feature maps. The feature map accentuates the unique features of the original data. The convolutional layer operates in a very different way compared to the other neural network layers. This layer does not employ connection weights and a weighted sum. Instead, it contains filters [29]. Every convolutional layer is connected to a rectified linear unit (ReLU) activation function, described in Equation (11). ReLU can remove the back propagation vanishing gradient problem during training and reduce the training time [30].

(11) y=max(0,x)

After each convolutional layer, a max-pooling layer is added. The max-pooling layer uses the maximum value in the filter with a specified size and then, conducts subsampling [31] Then, four fully connected layers (FCL) are added to the model, where the last one uses a sigmoid activation function for the classification of the output into the two possible classes (depressed and not depressed).

Generally, a CNN becomes too dependent on training data, which can cause “over-fitting” [32] To solve this problem, a regularization method based on dropout is added to each 2D-CNN layer, using a probability of 50%.

### 2.4. Classification Analysis

For the classification stage, the 2D-CNN and Deep Neural Network (DNN) is used to detect depressive episodes based on the new dataset generated in the second stage. This way we can compare whether there is a difference between the results obtained from one neural network and another. In this way, it will be possible to verify if there are indeed significant results for the implementation of the proposed 2D-CNN. Notably, the application of convolutional networks (CNN) has significantly improved the accuracy of human emotion recognition [33]. However, most efforts have focused on image processing and not on other data that may also be of great relevance even for mood change detection. According to Albawi et al. [34] a CNN is mathematical linear operation between matrixes called convolution. CNN have multiple layers, including convolutional layer, non-linearity layer, pooling layer and fully connected layer. The convolutional and fully connected layers have parameters, but pooling and non-linearity layers do not have parameters. The convolution for one pixel in the next layer is calculated according to Equation (12).

(12) $$net\left(t,j\right)=\left(x*w\right)\left[t,j\right]=\sum_{m}^{}\sum_{n}^{}x\left[m,n\right]W\left[t-m,j-n\right]$$

where net (t,j) is the output in the next layer, × is the input image and *w* is the kernel of filter matrix and ∗ is the convolution operation.

CNNs can be used for regression and classification task. Just by changing a few parameters, we do not necessarily need different networks. The main goal of a classification is to predict discrete (categorical) variables. The training phase builds a classifier by learning from a dataset and its associated class labels (depressed and non depressed). It is categorical, where each value serves as a category or class. In the test phase, the new data, whose class label is unknown, is used to identify the group to which they belong based on what is learned with the training data [35]. Prediction of continuous (numerical) variables is known as regression. In the case of depression, MADRS scores that show how severe a patient’s condition is can be predicted by CNN. The main difference is the choice of the loss function, in regression: mean square error (MSE), root MSE(L2), mean absolute error (L1), and huber loss, in classification problems: cross entropy, binary cross entropy, and hinge loss are typically used [36].

### 2.5. Validation

For the validation, five parameters are measured, the accuracy, sensitivity, specificity, receiver operating characteristic curve with area under the curve (ROC/AUC), and F1-Score.

Accuracy is used to have certain performance criteria, which refers to the degree to which the result of a calculation conforms to the correct value [23], and it is calculated using Equation (13).

(13) $$accuracy\left(1-error\right)=\frac{T_{P}+T_{N}}{C_{p}+C_{n}}$$

where $T_{p}$ are the true positives, $T_{n}$ are the true negatives, $C_{p}$ is the truly positive, and $C_{n}$ is the truly negative.

The accuracy of a classifier depends on the ratio between a number of targets correctly detected and all detected targets, known as precision, and the ratio between the number of targets correctly detected and all true targets, known as recall, shown in Equations (14) and (15).

(14) $$precision=\frac{T_{p}}{T_{P}+F_{P}}$$

(15) $$recall=\frac{T_{P}}{T_{p}+F_{N}}$$

where $F_{P}$ is false positive and $F_{N}$ is false negative.

Since precision and recall are both necessary for evaluating the detection capabilities of an algorithm, it is convenient to find a single measure that considers both of them, as the F1-Score, which is the harmonic mean of the precision and recall [37]. The F1-Score can be calculated with Equation (16).

(16) $$F_{1}=\frac{precision\cdot recall}{precision+recall}$$

The sensitivity refers to the proportion of true positives that are correctly identified by test [38], and it is calculated using Equation (17).

(17) $$Sensitivity\left(1-\beta \right)=\frac{T_{p}}{C_{p}}$$

The specificity is the portion of negative samples that are correctly classified [39], and it is calculated with Equation (18).

(18) $$Specificity\left(1-\propto \right)=\frac{T_{n}}{C_{n}}$$

ROC has been widely used to measure or visualize a classifier’s performance in conjunction with the AUC value to select a suitable operating point, called the decision threshold [40]. The simplest way to calculate the AUC is through trapezoidal integration, show in Equation (19).

(19) $$AUC=\sum_{i}^{}\left(1-\beta_{i}\cdot \Delta \alpha \right)+\frac{1}{2}\left[\Delta \left(1-\beta \right)\cdot \Delta \alpha \right]$$

where Δ(1−β) = $\left(1-\beta_{i}\right)-\left(1+\beta_{i-1}\right)$ and $\Delta \alpha =\alpha_{i}+\alpha_{i-1}$.

A confusion matrix is also calculated for the evaluation. The confusion matrix is typically used in machine learning to evaluate, and visualize the behavior of models in supervised classification contexts. It is a square matrix in which the rows represent the actual class of the instances, and the columns represent their predicted class. We are handling a binary classification task, and then the confusion matrix is a 2 × 2 that reports the number of true positives, true negatives, false positives, and false negatives [41].

All the experiments of this work are carried out in “python 3.7.10” in the framework jupyter notebook, a web application for creating and sharing computational documents. It offers a simple, streamlined, and document-centric experience [42].

## 3. Results

In this section, we show the results obtained.

**Figure 2:**
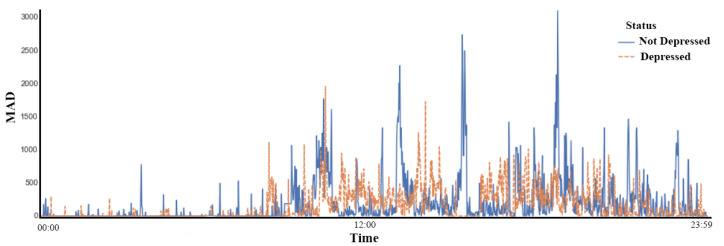
Example of samples collected with Actiwatch of a not depressed and a depressed subject of the Depresjon database.

Initially, In Figure 2 as can be seen, there is a notable difference between the two classes, where the decrease in movement among depressed people is evident. These data were taken for each of the 55 subjects of study during a week where each day was represented as an individual observation, obtaining a number of 385 observations (55 × 7).

In the second stage, of a total of 385 observations initially obtained from the dataset for analysis and processing, 70% (269) is used for balancing classes and to train the model, and 30% (116) remain for testing.

**Figure 3:**
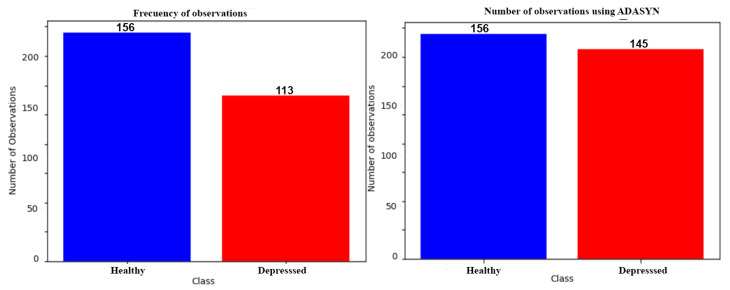
Graph of the number of observations. On the left side is the number of observations before class balancing and on the right side is the number of observations obtained using ADASYN.

Figure 3 presents a graph that allows observing the number of observations before (left side) and after (right side) applying the class balance. A total of 301 observations were obtained, where 156 are from healthy subjects and 145 from condition subjects. It is common to face the problem of severely unbalanced data. If classification analysis had been carried out with an unbalanced dataset, the model performance may be very poor, and conversely with balanced data it may be accurate [43].

**Figure 4:**
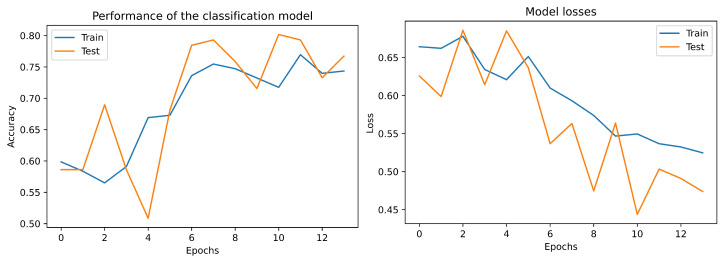
Loss and accuracy over the train and test data in the 2D-convolutional neural network proposed.

To measure the performance of the proposed neural network, it is necessary to establish that, according to Phatar et al. [44], for a perfect model validation, loss should be similar or slightly higher than the training loss. If the validation loss is lower than the training loss, then the model is underfit and it should be trained for more number of epochs. For our model, the above condition was satisfied at the 14th epoch, as can be seen in Figure 4, where the validation loss follows same pattern as the training—being slightly higher.

**Figure 5:**
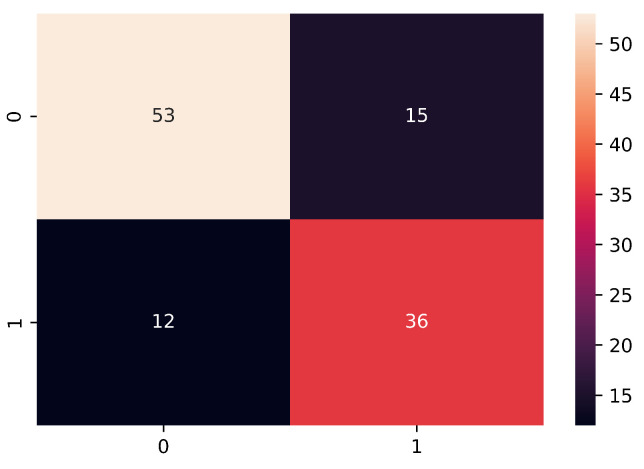
The confusion matrix. On the upper left side, true negatives (TP), on the upper right side, the false positives (FP), on the lower left side false negatives (FN) and on the lower right side, the true positives (TP): 1 is the number of observations per subject with depression and 0 is the number of observations per healthy subject.

For the development of the classification model, where a simple CNN was used, the accuracy performance obtained was of 76.72% and the F1-score of 72.72%. In Figure 5 we present the confusion matrix obtained through the classification of subjects using the test data.

**Figure 6:**
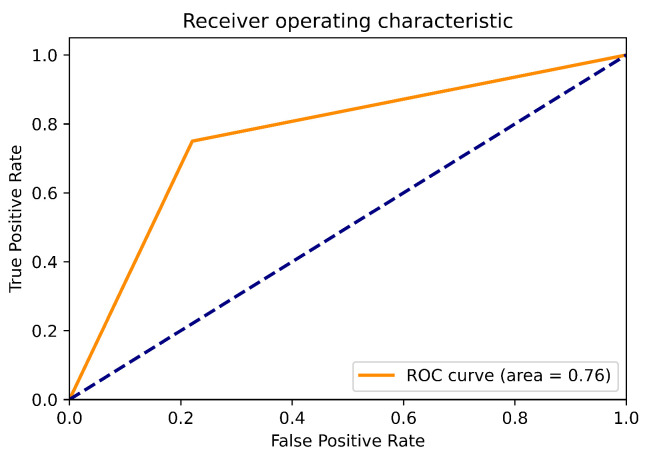
ROC curve obtained from the classification analysis based on 2D-CNN.

In Figure 6 the ROC curve obtained from the evaluation of the classification model with its respective AUC value can be observed The sensitivity and specificity values obtained were 0.75 and 0.77, respectively.

In the case of performing processing on an unbalanced dataset to obtain a classification model with a similar accuracy of 76.72%, this would not mean that the classifier would have the same capabilities to identify one subject from another. This is demonstrated below.

**Figure 7:**
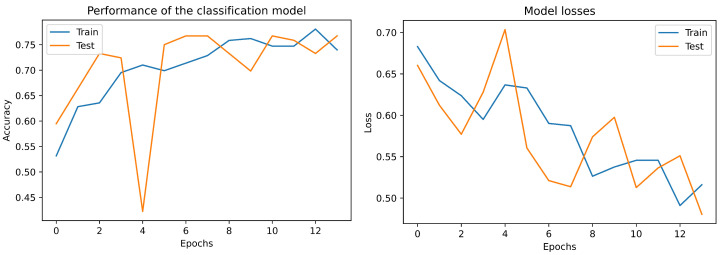
Loss and accuracy over the train and test data in the 2D-Convolutional neural network proposed with unbalanced data.

First, Figure 7 shows that the model has no overfitting problems, i.e., the model works perfectly on the training set and also on the test data.

**Figure 8:**
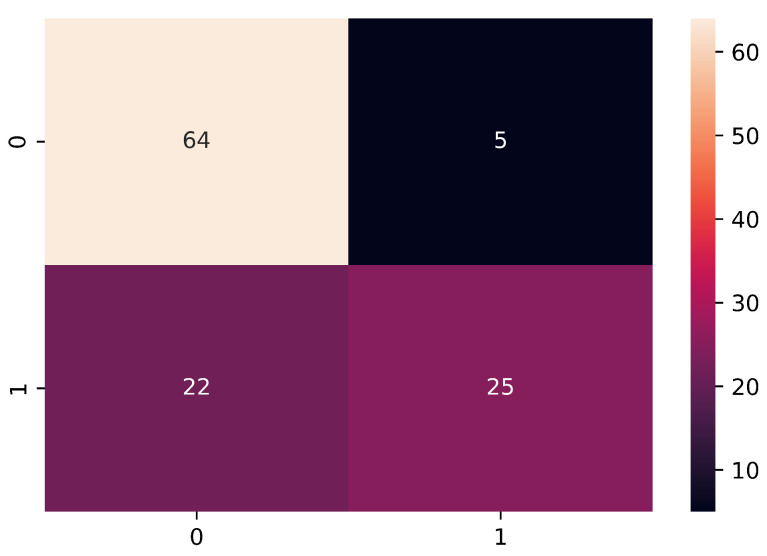
The confusion matrix obtained through unbalanced data processing.

However, the problem lies in the results obtained for sensitivity and specificity, where 0.53 and 0.92, respectively, were obtained. This means that the classification model is more susceptible to detecting more cases of true negatives as well as false positives, due to the higher number of observations of healthy subjects, and this can also be confirmed in the confusion matrix shown in Figure 8.

Based on these experimental results, we can say that to generate an optimal, efficient, and objective classification model, class balancing is essential in this case to improve the performance of the classification analysis.

## 4. Discussion

In Table 2, a comparison between a deep neural network with 1 input layer, 15 dense layers, and his Relu functions, 16 dropout layers, and 1 output layer with softmax function, was trained with the same number of epochs, batch size, and number of observations as the 2D-CNN (the structure of this deep neural network was proposed for comparative purposes only). In addition, we take the model proposed by Frogner et al. [25], based on a convolutional neural network (CNN) and results obtained in the work of Jakobsen et al. [27], where we analyze activity patterns in depressed patients and healthy controls with machine learning and deep learning.

The two neural networks proposed in this research work can be considered to have slightly similar results, where with a reduced number of observations for each test subject, an accuracy of 0.76 was obtained in the case of the 2D-CNN and 0.72 in the DNN. It should be noted that accuracy is a great measure, but only when you have symmetric datasets (false negatives and false positives are equals or close equals). Also, false negatives and false positives are similar. In this case, the test set is not symmetric, therefore it is appropriate to consider the F1 score as a validation metric for unequal class distribution. Based on this, it can be pointed out that 2D-DNN still has a better capacity for depression recognition, given that it has an F1-Score of 0.72 and DNN of 0.69, as shown in Table 2, but examining the results of sensitivity and specificity DNN is more susceptible than classifying more TN (47) to TP (36). ven the rate of FN (23) is very high, considering that they are cases that should be considered delicate, since mental health is a very serious problem today, unlike 2D-CNN, which classifies both cases almost to the same extent, which would be the ideal.

Results obtained in previous investigations, such as the one shown in the investigation by Frogner et al. [25], where the number of observations used for model training obtained an accuracy of 0.70, shows that the complexity of the methodology is much greater than the proposals in this work.

On the other hand, the results obtained in the work of Jakobsen et al. [27] are not very significant when implementing convolutional neural networks. This could be derived from the lack of predictors or features to improve their performance.

In our methodology, accuracy can be improved with new input values and a greater number of observations, but it would be important to consider not increasing the processing time as well as computational cost, since one of the main contributions of this research is the simplicity of experimentation and development.

A simple convolutional neural network capable of working and retraining in real time for a single device without great processing capacity that can improve its results with the input of new data collected within a week would allow daily monitoring of patients.

Although there are some other works with different techniques and the same approach, some of them do not meet the problem of adaptability for different types of users for not working immediately. This means that to generate a depression classification model, it is necessary to generate a dataset of different test subjects who are in different uncontrolled situations and finally go to a stage of analysis and processing of all the information collected.

Nevertheless, the results presented in the table above were obtained by applying different settings, methodologies, and validation metrics that may cast doubt on the effectiveness of the proposed method. For this, it was necessary to perform 3-fold cross-validation tests and objectively compare the results.

**Figure 9:**
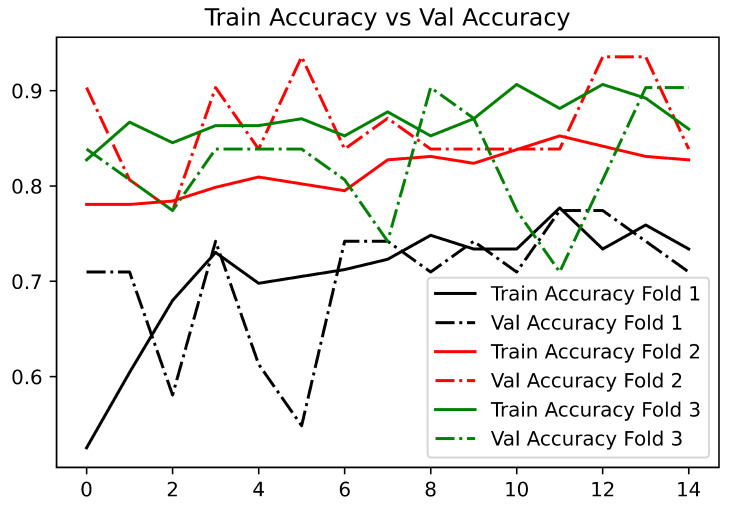
Training and validation accuracy using 14 epochs in 2D-CNN with 3 fold cross-validation.

The performance of the 2D-CNN model during each epoch in each k-fold can be observed in Figure 9. The same procedure was followed as in the papers discussed in this section. We split the dataset into a train and test. Then, we generated three folds containing training and validation parts to train a model for each fold

Table 3 shows the values obtained by 2D-CNN in each of the folds and those achieved in the work of Frogner et al. [25]. If the accuracy was still high and the loss was still low, the model would have a good chance of doing correct classifications on unseen data.

In the case of Jakobsen et al. [27], a replication of their experimental process is omitted for the comparison of results, because it uses statistical features as the main source of information.

Even though each of the k-folds has a considerable difference in accuracy between the state of art and this study, the robustness of our results is promising and demonstrates their efficiency in maintaining and even improving their performance on unseen data (outside the data used in cross-validation), as shown in Table 4, which includes macro average (averaging the unweighted mean per label) and weighted average (averaging the support weighted mean per label).

## 5. Conclusions

This research proposed a methodology composed of a series of steps, consisting of the generation of a new dataset from the capture of motor activity using a simple and minimally invasive device, class balancing, development of a classification model, and validation. The main objective was to find a simple neural network architecture that could objectively detect depressed subjects.

Despite the fact that the results are not yet concrete for their implementation in real life, this is a very important starting point, as the data obtained through the motor activity measurements allowed to create of a model capable of distinguishing between a depressive and a not depressive subject. Evaluation of the performance through different metrics allowed us to know the scope that can be reached to identify episodes of depression in an objective and less complex way, which can reduce costs regarding its commercialization or commissioning in the future. Although there are different ways to attack the problem, a solution could be in constant monitoring that allows the problem to be identified prematurely. Here it is demonstrated that by implementing artificial intelligence techniques it is possible to create tools that identify possible cases of depression.

It is important to note that the reduced number of subjects allows the model to obtain significant results with a low computational cost. However, the number of data extracted in real-time from a subject must complement the period of one week from a device that extracts MAD to retrain 2D-CNN efficiently. For this, it is also necessary that the base dataset is balanced—that there is the same number of observations for each healthy subject and for each subject with depression—in order not to have overfitting problems that make it difficult to identify depression. An important consideration is to improve the performance of the model from the increase of observations per subject and the reduction of the time-lapses to identify this and other mental disorders.

These types of neural networks models can be used to analyze depression episodes in subjects, where the professional on mental health can use the data collected with smart devices that record motor activity during a period and give them to the model to be classified. The result of this classification can be used as support for the final diagnosis of the subject.

## Figures and Tables

**Table 1:** 2D-CNN architecture used in our research (i.e., Conv, ReLU, Pool, and FCL mean convolutional layer, rectified linear unit, max pooling layer, and fully connected layers, respectively).

Layer Type	Number of Filters	Size of Feature Map	Size of Kernel	Number of Stride	Number of Padding
Image Input Layer	30 (Height) × 48 (Width)				
Conv2d_1	48	26 × 44 × 48	3 × 3	1 × 1	1 × 1
ReLU_1	1	26 × 44 × 48			
Pool_1	48	13 × 22 × 48	3 × 3	2 × 2	1 × 1
Conv2d_2	48	11 × 20 × 48	3 × 3	1 × 1	1 × 1
ReLU_2	1	11 × 20 × 48			
Pool_2	48	5 × 10 × 48	3 × 3	2 × 2	1 × 1
Conv2d_3	48	3 × 8 × 48	3 × 3	1 × 1	1 × 1
ReLU_3	1	3 × 8 × 48			
Pool_3	48	1 × 4 × 48	3 × 3	2 × 2	1 × 1
1st FCL		900 × 1			
ReLU_4		900 × 1			
Pool_4		900 × 1			
2nd FCL		300 × 1			
ReLU_5		300 × 1			
Pool_5		300 × 1			
3rd FCL		100 × 1			
ReLU_5		100 × 1			
Pool_5		100 × 1			
4th FCL		2 × 1			
Sigmoid		2 × 1			
Output layer		2 × 1			

**Table 2:** Results obtained from proposed work from past investigations that also use convolutional neural networks to detect depression. N/M: the research does not mention the result obtained.

	Accuracy	Sensitivity	Specificity	AUC	F1-Score
2D-CNN (Our proposed work)	0.76	0.75	0.77	0.76	0.72
Deep Neural Network	0.72	0.61	0.83	0.73	0.69
Frogner et al. [25]	0.71	0.60	0.70	N/M	0.70
Jakobsen et al. [27]	0.57	0.18	0.78	N/M	N/M

**Table 3:** Loss and accuracy of 3-fold cross-validation obtained in previous works and our proposed work.

	1-Fold	2-Fold	3-Fold	Mean
Accuracy (2D-CNN)	0.70	0.83	0.90	0.81
Loss (2D-CNN)	0.48	0.34	0.27	0.36
Accuracy (Frogner et al. [25])	0.98	0.98	0.98	0.98
Loss (Frogner et al. [25])	0.06	0.07	0.06	0.063

**Table 4:** Summary results of the precision, recall, F1-Score for each class obtained from the proposed work with unseen data.

	Precision	Recall	F1-Score	Support
Not Depressed	0.84	0.79	0.81	72
Depressed	0.69	0.75	0.72	44
Accuracy			0.78	116
Macro avg	0.76	0.77	0.77	116
Weighted avg	0.78	0.78	0.78	116

## Data Availability

The dataset can be accessed via: http://datasets.simula.no/depresjon/, accessed on 20 January 2022, or directly downloaded from https://doi.org/10.5281/zenodo.1219550, accessed on 20 January 2022 and contains the following: Two folders, whereas one contains the data for the controls and one for the condition group. For each patient we provide a csv file containing the actigraph data collected over time. The columns are: timestamp (one minute intervals), date (date of measurement), activity (activity measurement from the actigraph watch).
